# High possibility of hepatocarcinogenesis in HBV genotype C1 infected Cambodians is indicated by 340 HBV C1 full-genomes analysis from GenBank

**DOI:** 10.1038/s41598-019-48304-z

**Published:** 2019-08-21

**Authors:** Channarena Chuon, Kazuaki Takahashi, Junko Matsuo, Keiko Katayama, Chikako Yamamoto, Ko Ko, Sirany Hok, Shintaro Nagashima, Sheikh Mohammad Fazle Akbar, Junko Tanaka

**Affiliations:** 1 Department of Epidemiology, Infectious Diseases Control and Prevention, Graduate School of Biomedical and Health Sciences, Hiroshima University, Minami-ku, Japan; 2 Ministry of Heath, Phnom Penh, Cambodia; 3 Department of Pathology, Proteo Science Center, Ehime University, Ehime, Japan

**Keywords:** Epidemiology, Hepatitis B

## Abstract

Approximately 75% of hepatocellular carcinomas (HCC) occur in Asia; core promoter mutations are associated with HCC in HBV genotype C, the dominant genotype in Cambodia. We analyzed these mutations in Cambodian residents and compared them with HBV full genomes registered in GenBank. We investigated the characteristics of 26 full-length HBV genomes among 35 residents positive for hepatitis B surface antigen in Siem Reap province, Cambodia. Genotype C1 was dominant (92.3%, 24/26), with one case of B2 and B4 each. Multiple mutations were confirmed in 24 Cambodian C1 isolates, especially double mutation at A1762T/G1764A in 18 isolates (75.0%), and combination mutation at C1653T and/or T1753V and A1762T/G1764A in 14 isolates (58.3%). In phylogenetic analysis, 16 of 24 isolates were located in the cluster with Laos, Thailand, and Malaysia. In 340 GenBank-registered C1 strains, 113 (33.2%) had combination mutation amongst which 16.5%, 34.2%, and 95.2% were found in ASC, chronic hepatitis, and liver cirrhosis (LC)/HCC respectively (*P* < 0. 001). Mutations were abundantly found in 24 Cambodian C1 isolates, and 340 C1 strains from GenBank showed mutation in genotype C1 brings high possibility of LC/HCC occurrence. Therefore, we suggest that Cambodian people infected with HBV genotype C1 have high possibility of hepatocarcinogenesis.

## Introduction

Liver cancer is the second most common cause of cancer-related deaths worldwide and was estimated to cause nearly 746,000 deaths in 2012 (9.1% of all cancer-related deaths that year)^1^. Hepatocellular carcinoma (HCC) accounts for more than 90% of cases of primary liver cancer^2^ and chronic hepatitis B virus (HBV) infection is the leading cause of liver diseases evolving into liver cirrhosis and HCC^3,4^. Moreover, 60% of HCC is associated with HBV, whereas 20% is related to hepatitis C virus^5^ in Africa and Asia. The majority of all cases of HCC worldwide are found in the Asian Pacific region, and approximately 75% of liver cancer cases occur in Asia^5^.

HBV genotypes B and C are dominant in Asia, and genotype C plus core mutations in the HBV genome are associated with higher risk of HCC than genotypes A, B, and D^6–8^. In addition, double mutation in the basal core promoter (A1762T/G1764A) of HBV genotype C was commonly found as an independent risk factor for the development of HCC^9–11^. HBV gene mutations were reported in 1998 by Takahashi^12^ in many parts of the gene, as pre-S deletion and multi-site mutations in the core promoter and at core protein aa 130 are associated with HCC. Mutations at C1653T and/or T1753V and A1762T/G1764A in Enhancer II/basal core promoter were also reported to be associated with HCC in 1999 compared with other liver disease statuses^13^. Subsequently, many reports confirmed these mutations^14,15^. The combination mutation involving the double mutation at A1762T/G1764A and mutation at C1653T and/or T1753V^14^ has now been shown to be a risk factor for HCC occurrence^16–18^. Prompt anti-viral treatment is proposed for such patients to prevent HCC^18^.

The Kingdom of Cambodia is one of 37 countries located in the Western Pacific Region and has been reported to be highly endemic for HBV infection. In Cambodia, liver cancer is the second leading cause of cancer-related deaths and is responsible for 21.5 of every 100,000 deaths annually^19^. From 2010 to 2014, we conducted a pilot sero-epidemiological survey on hepatitis virus infection among the general population and elementary school students in Siem Reap province, Cambodia in cooperation with the Ministry of Health in Cambodia^20–22^. In our previous survey, we found that the prevalence of hepatitis B surface antigen (HBsAg) was 4.6% and that genotype C was dominant among adults in Cambodia^21^. One report showed that genotype C1 accounted for 66.7% of cases and that genotype B1 was identified in 12 isolates from Cambodia^23^.

In this study, we performed a genetic analysis of HBV carriers among Cambodian residents by full-length genomic sequencing to clarify the characteristics of HBV genomes and to predict the occurrence of HCC. We used 340 full genomes of genotype C1 registered in GenBank for comparison.

## Results

### Participants in Cambodia

In total, 626 participants (254 men and 372 women, age range: 7–90 years as of 2014, average age: 38.3 + 16.3 years) were recruited in our survey. The participants were from the general population of Chrey village (n = 333), Sasar Sdam commune (n = 55), Krabei Riel commune (n = 189), and Rohal village (n = 49)^21^.

### Prevalence of HBV infection in 626 participants

Table 1 shows the age/sex-specific seroprevalence of HBsAg, anti-HBs, and anti-HBc among 626 participants. The prevalence of HBsAg, anti-HBs, and anti-HBc were 5.6% (95% CI: 3.8–7.4), 28.0% (95% CI: 24.4–31.5), and 35.3% (95% CI: 31.6–39.0), respectively. The prevalence rate of HBsAg in men (7.9%; 95% CI: 4.6–11.2) was higher than that in women (4.0%; 95% CI: 2.0–6.0).

**Table 1:** The Age-sex specific prevalence of hepatitis B infection among 626 general populations in Siem Reap province, Cambodia.

		N	HBsAg POSITIVE		Anti-HBs POSITIVE		Anti-HBc POSITIVE	
			n (%)	95%CI	n (%)	95%CI	n (%)	95%CI
Total		626	35 (5.6)	3.8–7.4	175 (28.0)	24.4–31.5	221 (35.3)	31.6–39.0
Age group (years)	7–19	88	2 (2.3)	0–5.4	10 (11.4)	4.7–18.0	5 (5.7)	0.8–10.5
	20–29	118	3 (2.5)	0–5.4	19 (16.1)	9.5–22.7	25 (21.2)	13.8–28.6
	30–39	136	11 (8.1)	3.5–12.7	38 (27.9)	20.4–35.5	51 (37.5)	29.4–45.6
	40–49	124	7 (5.6)	1.6–9.7	41 (33.1)	24.8–41.3	56 (45.2)	36.4–53.9
	50–59	85	8 (9.4)	3.2–15.6	37 (43.5)	33.0–54.1	49 (57.6)	47.1–68.2
	60–90	75	4 (5.3)	0.2–10.4	30 (40.0)	28.9–51.1	35 (46.7)	35.4–58.0
Male		254	20 (7.9)	4.6–11.2	78 (30.7)	25.0–36.4	105 (41.3)	35.3–47.4
Age group (years)	7–19	40	2 (5.0)	0–11.8	6 (15.0)	3.9–26.1	4 (10.0)	0.7–19.3
	20–29	56	2 (3.6)	0–8.4	10 (17.9)	7.8–27.9	15 (26.8)	15.2–38.4
	30–39	48	6 (12.5)	3.1–21.9	15 (31.3)	18.1–44.4	26 (54.2)	40.1–68.3
	40–49	61	4 (6.6)	0.3–12.8	24 (39.3)	27.1–51.6	29 (47.5)	35.0–60.1
	50–59	31	5 (16.1)	3.2–29.1	13 (41.9)	24.6–59.3	22 (71.0)	55.0–86.9
	60–90	18	1 (5.6)	0.0–16.1	10 (55.6)	32.6–78.5	9 (50.0)	26.9–73.1
Female		372	15 (4.0)	2.0–6.0	97 (26.1)	21.6–30.5	116 (31.2)	26.5–35.9
Age group (years)	7–19	48	0 (0.0)	0–7.7	4 (8.3)	0.5–16.2	1 (2.1)	0–6.1
	20–29	62	1 (1.6)	0–4.7	9 (14.5)	5.7–23.3	10 (16.1)	7.0–25.3
	30–39	88	5 (5.7)	0.8–10.5	23 (26.1)	17.0–35.3	25 (28.4)	19.0–37.8
	40–49	63	3 (4.8)	0–10.0	17 (27.0)	16.0–37.9	27 (42.9)	30.6–55.1
	50–59	54	3 (5.6)	0–11.7	24 (44.4)	31.2–57.7	27 (50.0)	36.7–63.3
	60–90	57	3 (5.3)	0–11.1	20 (35.1)	22.7–47.5	26 (45.6)	32.7–58.5

HBsAg: hepatitis B surface antigen, Anti-HBs: Hepatitis B surface antibody, anti-HBc: Hepatitis B core antibody, 95% CI: 95% Confidence Interval.

### Phylogenetic analysis and genotyping of 26 HBV infected residents in Cambodia

**Figure 1:**
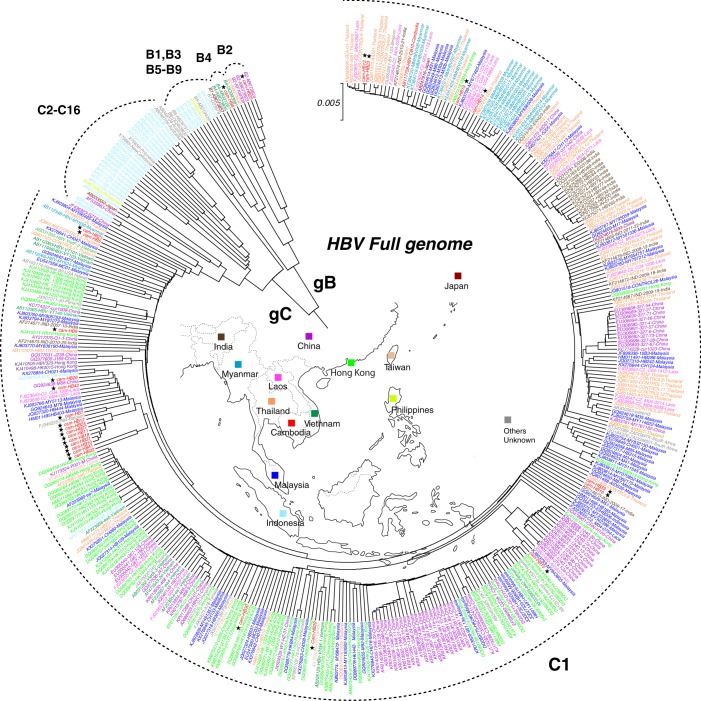
Phylogenetic tree generated using the UPGMA method with 26 Cambodian isolates. The phylogenetic tree was constructed using the 26 isolates with HBV full genomes in this study, and HBV genotypes B1–B9 strains, and C1–C16 strains registered in GenBank by the UPGMA method. The analysis involved 416 complete nucleotide sequences. Evolutionary analyses were conducted using MEGA7. Our Cambodian samples are marked with stars. Other strains are shown according to colour based on location in Southeast Asia at the centre of the phylogenetic circle.

Phylogenetic tree analysis with the Unweighted Pair Group Method with Arithmetic Mean (UPGMA) method of the full genomes showed that 24 of 26 isolates belonged to genotype C1, and one belonged to genotype B2 and B4 each (Fig. 1). In Fig. 1, an Asian map in the phylogenetic circle is shown with all isolates in the same colour of their country indicated.

**Figure 2:**
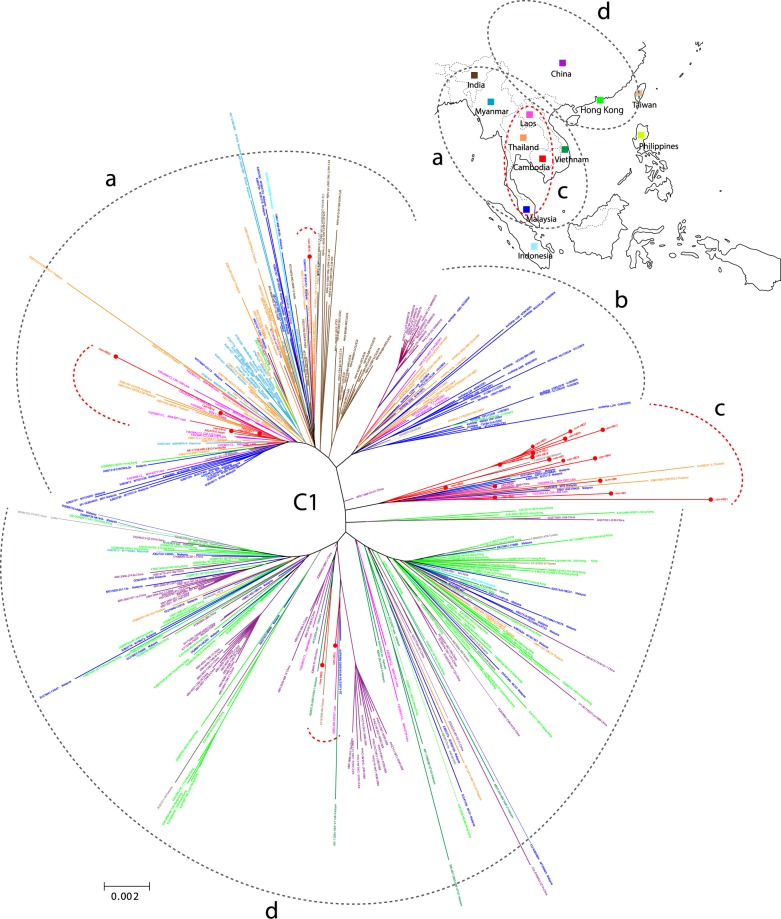
Phylogenetic tree generated using the neighbour-joining method with 24 genotype C1 Cambodian isolates from this study and 340 HBV C1 strains registered in GenBank. The analysis involved 364 HBV genotype C1 complete nucleotide sequences, which were separated into over 10 different clusters, but primarily clusters (**a**–**d**). The 24 isolates obtained here were categorized into three clusters (**a**,**c**, and **d**). Cluster (**a**) contained six Cambodian isolates that were close to strains from Myanmar, Laos, Thailand, and Malaysia, and cluster (**d**) contained two Cambodian isolates that were close to strains from China or Hong Kong. Cluster **c** was composed of mainly 16 Cambodian isolates and other strains from Laos, Thailand, and Malaysia, spreading into a narrow area of the Southeast Asian countries.

Next, phylogenetic tree analysis with the neighbour-joining method was used to compare 24 genotype C1 isolates with 340 HBV genotype C1 strains from many countries registered in GenBank. The results showed that the sequences were separated into more than ten clusters (Fig. 2), and mainly into four clusters (clusters **a–d**). Twenty-four Cambodian isolates existed in clusters **a**, **c**, and **d**. Cluster **a** was composed of strains from India and Southeast Asia (including Myanmar, Laos, Thailand, Malaysia, Cambodia) and six isolates from this study. Cluster **c** was composed of mainly sixteen Cambodian isolates from this study and some strains from Laos, Thailand, and Malaysia spreading over a narrow area of Southeast Asian countries (see map in Fig. 2). Cluster **d** was primarily composed of strains from China and Hong Kong, with some strains from Thailand, Malaysia, Laos, Vietnam, and two Cambodian isolates obtained in this study.

### Profiles and mutations of HBV full-length genome sequencing of 26 HBV-infected residents in Cambodia

Of the 26 HBV carriers, 13 were men, and 13 were women (13–70 years old) (Table 2). The mean age of the Cambodia cohort was 42 ± 14 years. Eighteen of the HBV carriers (69.2%) were residents of Chrey village, 7 (26.9%) were from Sasar Sdam Commune, and 1 was from Krabei Riel Commune. They were all positive for HBsAg and anti-HBc, but negative for anti-HBs.

**Table 2:** Profiles and mutations of 26 HBV DNA full-genome sequences.

Isolate Name	Age/Sex	Resi-dence	HBsAg RPHA titer	Anti-HBs	Anti-HBc	HBeAg/HBeAb	Genotype	Cluster	Length (bp)	PreS Deletion	Core Promoter				PreC	Core	Note
											1613	1653	1753	1762/1764	W28 stop	P130/T/A/I	
Cam-HB 25	15/M	CV	>12	−	+	+/−	B2		3215								
Cam-HB 8	52/F	SC	4	−	+	−/+	B4		3215		(+)				(+)		
Cam-HB 13	23/M	CV	>12	−	+	+/−	C1	a	3215								
Cam-HB 14	30/M	CV	5	−	+	−/+	C1	a	3215						(+)		
Cam-HB 16	47/M	CV	9	−	+	−/+	C1	a	3213	(+)	(+)					(+)	1 bp insertion in PreC
Cam-HB 19	38/F	CV	9	−	+	−/+	C1	a	3215					(+)			
Cam-HB 23	40/M	CV	5	−	+	−/+	C1	a	3215								
Cam-HB 33	70/F	KC	6	−	+	ND	C1	a	3179	(+)		(+)		(+)			
Cam-HB 15	42/F	CV	5	−	+	+/−	C1	d	3215		(+)	(+)		(+)		(+)	PreS2 Start codon missing
Cam-HB 29	34/M	CV	>12	−	+	+/−	C1	d	3215								
Cam-HB 5	63/F	SC	5	−	+	−/+	C1	c	3215			(+)		(+)	(+)		
Cam-HB 7	53/M	SC	10	−	+	−/+	C1	c	3215				(+)	(+)	(+)	(+)	
Cam-HB 9	53/M	SC	8	−	+	−/+	C1	c	3215		(+)				(+)		
Cam-HB 10	36/F	CV	>12	−	+	−/+	C1	c	3215		(+)	(+)		(+)		(+)	
Cam-HB 11	46/F	CV	9	−	+	−/−	C1	c	3215				(+)	(+)	(+)	(+)	
Cam-HB 12	45/M	CV	10	−	+	−/+	C1	c	3216				(+)	(+)		(+)	1 bp insertion in PreC
Cam-HB 18	68/F	CV	7	−	+	−/+	C1	c	3215				(+)	(+)	(+)	(+)	
Cam-HB 21	58/M	SC	>12	−	+	−/+	C1	c	3191	(+)			(+)	(+)		(+)	
Cam-HB 22	34/M	CV	8	−	+	−/+	C1	c	3215				(+)	(+)		(+)	
Cam-HB 24	33/M	CV	>12	−	+	−/+	C1	c	3215				(+)	(+)		(+)	
Cam-HB 26	46/F	CV	11	−	+	+/−	C1	c	3215			(+)		(+)			
Cam-HB 27	33/F	CV	>12	−	+	−/+	C1	c	3215				(+)	(+)			
Cam-HB 30	56/F	CV	12	−	+	−/+	C1	c	3215				(+)	(+)			
Cam-HB 31	17/M	CV	>12	−	+	+/−	C1	c	3215					(+)			
Cam-HB 41	30/F	SC	>12	−	+	−/+	C1	c	3179	(+)	(+)			(+)		(+)	
Cam-HB 42	35/F	SC	12	−	+	+/−	C1	c	3215					(+)			

**Figure 3:**
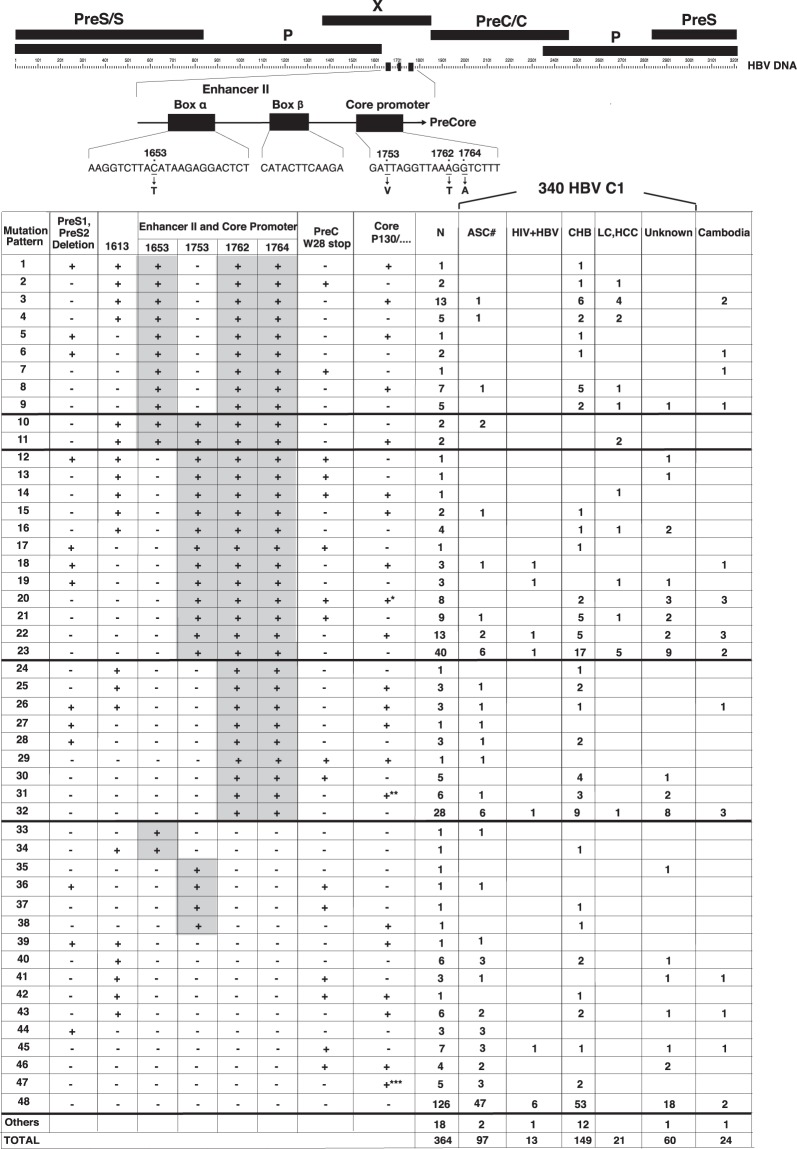
Mutations focused at the core promotor were classified into 47 patterns that were confirmed in 364 HBV genotype C1 full genomes including the 24 isolates in this study. *Threonine (T)/Leucine (L), **Isoleucine (I)/Leucine (L)/Threonine (T), ***Histidine (H)/Isoleucine (I)/Glutanine (Q)/Threonine (T). ASC#: asymptomatic carrier including blood donor general population and occult hepatitis B infection, HIV + HBV: Human immunodeficiency virus and HBV co-infected patients, CH: patients with Chronic Hepatitis, LC/HCC: patients with Liver Cirrhosis or Hepatocellular Carcinoma. The registered 340 HBV genotype C1 strains and 24 isolates were analysed and classified by their mutations. The map of the whole region and genome is shown in upper case. C1653T, T1753V, A1762T/G1764A exist in Enhancer II and the Basal Core Promoter. The classifications were quite detailed to show the distribution. Patterns show assortments of each mutation; pattern 47 means that the HBV gene has no mutations at the special-focused point in our study, and the pattern “others” means that the HBV gene has some mutations but not at the focused point.

M: male, F: female, CV: Chrey Village, SC: Sasar Sdam Commune, KC: Krabei Riel Commune, HBsAg: Hepatitis B surface antigen, anti-HBs: antibody to Hepatitis B surface antigen, anti-HBc: antibody to Hepatitis B core antigen, HBeAg: Hepatitis B e antigen, HBeAb: Hepatitis B e antibody, ND; not done, RPHA: Reversed Passive Hemagglutination. Isolate characteristics are shown, including the genotype, HBsAg, anti-HBc, HBeAg status, full length, presence or absence of Pre-S deletion and mutations with their profiles such as gender and the place of living. All 26 Cambodian samples were negative for anti-HBs (antibody to Hepatitis B surface antigen). For genotype C1 isolates, clusters are shown as letters in the phylogenetic tree in Fig. 3. For point mutations, G1613A, C1653T, T1753V, A1762T/G1764A, precore G1896A for a stop codon mutation and aa 130 in core region are shown. Cam-HB 16 and Cam-HB 12 had one base pair insertion. In the 24 genotype C1 isolates, many mutations were found, and especially the double mutation at A1762T/G1764A was recognized in the 18 isolates (18/24, 78.3%).

Among the 26 isolates, the nucleotide lengths were within 3179–3216 bp (Table 2).

In the 24 genotype C1 isolates, many mutations were found. The double mutation at A1762T/G1764A was recognized in 18 of the isolates in genotype C1 (18/24 75.0% Table 2). Combination mutation was observed in 14 isolates (14/24 58.3%, 95% CI: 38.6–78.1). Of these, five at C1653T and A1762T/G1764A and nine at T1753V and A1762T/G1764A were recognized. Combination mutation at T1753V and A1762T/G1764A was most frequent. There was no solitary mutation at C1653T or T1753V.

Based on the phylogenetic analysis of genotype C1 (Fig. 2), among the 16 isolates from our study located in cluster **c**, 15 isolates had the A1762T/G1764A mutation and 12 isolates had combination mutation (Table 3). Mutation at G1613A was found in five isolates. Regarding the other mutations, there were four isolates with mutation at pre-S deletion (4/24) and 11 isolates at the Core P130 (11/24).

**Table 3:** Double mutation and combination mutation in 340 HBV genotype C1 strains retrieved from GenBank and 24 isolates Cambodian in the study.

Mutation Pattern	340 C1 strains in GenBank						24 Cambodian
	Sum of n (%)	ASC# n (%)	HIV+HBV n (%)	CH n (%)	LC/HCC n (%)	Unknown n (%)	Sum of n (%)
Double mutation pattern 1 to 32)	160 (47.1)	28 (28.9)	5 (38.5)	73 (49.0)	21 (100)	33 (55)	18 (75.0)
Combination mutation (pattern 1 to 23)	113 (33.2)*	16 (16.5)	4 (30.8)	51 (34.2)**	20 (95.2)	22 (36.7)	14 (58.3)
Only double mutation (pattern 24 to 32)	47 (13.8)	12 (12.4)	1 (7.7)	22 (14.8)	1 (4.8)	11 (18.3)	4 (16.7)
Others (pattern 33 to 48)	180 (52.9)	69 (71.1)	8 (61.5)	76 (51.0)	0	27 (45)	6 (25.0)
Total	340 (100)	97	13	149	21	60	24 (100)

In compared to 24 Cambodian, *(p = 0.0128, chi-squared = 6.203), **(p = 0.0241, chi-squared = 5.090), ASC#: Asymptomatic Carriers including Blood Donors, occult hepatitis B infection and general population, HIV + HBV: Human Immunodeficiency Virus with Hepatitis B Virus, CH: Chronic Hepatitis, LC/HCC: Liver Cirrhosis/Hepatocellular Carcinoma.

As shown in the table, 340 HBV genotype C1 strains retrieved from the GenBank and 24 isolates from Cambodian were summed up for double mutations. The liver disease status could not be known in 60 out of total 340 HBV genotype C1 strains retrieved from the GenBank. One hundred sixty strains, summed up the pattern 1 to 32 in the Fig. 3, had the double mutations at A1762T/G1764A in 340 C1 strains, and it was composed with 113 strains of combo mutation and 47 strains of only double mutations alone. There were 180 strains which did not have the double mutations in “Others” of pattern 33 to 48 in Fig. 3. The rate of double mutation was 47.0% and the rate of combo mutation was 33.2% in 340 genotype C1 strains. Both rates were higher in 24 Cambodian isolates than those in 340 genotype C1 strains.

For the isolates of genotype B2 and B4, only the genotype B4 isolate had a mutation at G1613A.

Seven of the 18 isolates that were negative for HBeAg had a stop codon at the precore region nt1896 (7/18, 38.9%). Two other isolates had a one base pair insertion at the precore region, which caused a frameshift in the precore protein.

### Mutations and liver disease status among 340 genotype C1 genomes in GenBank

After filtering by drawing phylogenetic trees several times with strains obtained by BLAST from NCBI, we finally extracted 340 genotype C1strains from GenBank.

Based on the presence or absence of the mutations of pre-S deletion, G1613A, C1653T, T1753V, A1762T/G1764A, Pre-C W28 stop codon, and P130, these 340 genotype C1 strains were classified in 48 patterns. They were then evaluated based on liver status obtained from the registered information or published papers (Fig. 3). In patterns 1 to 34 in Fig. 3, double mutation at A1762T/G1764A was confirmed in 160 strains (160/340, 47.1%), almost a half of the 340 strains (Table 3). Mutation patterns 1–23 in Fig. 3 represented combination mutation. In details, patterns 1 to 9 represented combination mutation at C1653T and A1762T/G1764A (32/340 9.4%), patterns 10 and 11 at C1653T or T1753V and A1762T/G1764A (4/340 1.25%), and patterns 12 to 23 at T1753V and A1762T/G1764A (77/340 22.6%). In patterns 24 to 32, 47 strains of double mutation at A1762T/G1764A were recognized (47/340 13.8%). In patterns 33 to 48, single mutation at G1613A, C1653T, T1753V, or Core P130 was observed in 18, 2, 4 and 18 strains, respectively (Supplementary Table 1).

**Figure 4:**
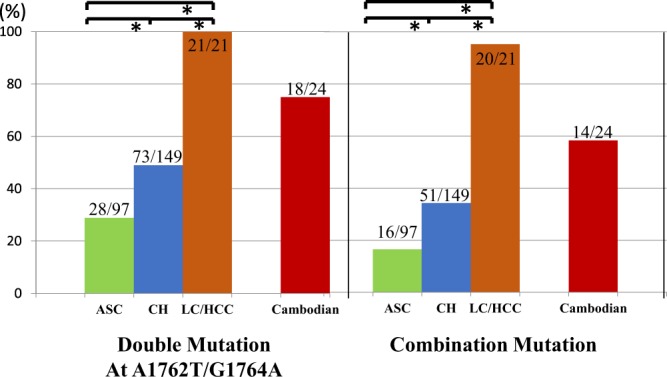
The rates of double mutation at A1762T/G1764A and combination mutation at C1653T and/or T1753V and A1762T/G1764A by liver disease status in 340 genotype C1 strains and Cambodian isolates. *P < 0.001. The rates of double mutation at A1762T/G1764A were raised significantly according to liver disease progression (among 3 groups *P* < 0.001; posthoc pairwise ASC vs CH *P* < 0.001, ASC vs LC/HCC *P* < 0.001, CH vs LC/HCC *P* < 0.001). The rates of combination mutation at C1653T or T1753V and A1762T/G1764A were also raised significantly according to liver disease progression (among 3 groups *P* < 0.001; posthoc pairwise ASC vs CH *P* < 0.001, ASC vs LC/HCC *P* < 0.001, CH vs LC/HCC *P* < 0.001). The rates of both double mutation and combination mutation in 24 Cambodian isolates were higher than those of ASC and CH in 340 genotype C1 strains.

In 340 C1 strains retrieved from GenBank, we investigated the associations between their liver disease status and special mutations (Table 3). The rates of retaining double mutation at A1762T/G1764A in ASC, patients with chronic hepatitis, and patients with LC/HCC were 28.9% (28/97), 49.0% (73/149), and 100% (21/21), respectively. The rates of double mutation were raised significantly according to liver disease progression (Among 3 groups, *p* < 0.001; posthoc pairwise ASC vs CH, *P* < 0.001, ASC vs LC/HCC, *P* < 0.001, CH vs LC/HCC, *P* < 0.001; Fig. 4). The rates of combination mutation in ASC, patients with chronic hepatitis, and patients with LC/HCC were 16.5% (16/97), 34.2% (51/149), and 95.2% (20/21), respectively. The rates of combination mutation at C1653T or T1753V and A1762T/G1764A were also raised significantly according to liver disease progression (Among 3 groups *P* < 0.001; posthoc pairwise ASC vs CH *P* < 0.001, ASC vs LC/HCC *P* < 0.001, CH vs LC/HCC *P* < 0.001; Fig. 4).

In patients with LC/HCC, the rates of Pre-S deletion, G1613A, and Core P130 mutation were 4.8% (1/21), 52.4% (11/21) and 38.1% (8/21) respectively. Strains with single mutation at G1613A, C1653T, T1753V, or Core P130 were not confirmed in patients with LC/HCC (Fig. 3).

## Discussion

Despite the high prevalence of HBV infection and high mortality from HCC in Cambodia, only few reports have been published on the molecular characterisation of HBV genomes in this country, and only three complete genomic sequences have been reported^23–25^. We performed complete genomic sequencing of 26 HBV carriers in Cambodia and found that the genotype of the 26 HBV carriers was genotype C1 dominant, consistent with a previous report in Cambodia^24^.

With phylogenetic tree analysis of 24 Cambodian genotype C1 isolates in this study and 340 genotype C1 strains registered in GenBank, we revealed the geographical distribution of HBV genotype C1 (Fig. 2). By the neighbour-joining method, these C1 strains were separated mainly into four clusters, with cluster **a** and cluster **d** being the largest. Cluster **a** was composed of strains from India and Southeast Asia and cluster **d** was composed of strains from China and Hong Kong. Sixteen of 24 Cambodian C1 isolates were located in cluster **c** with strains from Laos, Thailand, and Malaysia, neighboring countries to Cambodia.

Among 24 Cambodian C1 isolates, the double mutation at A1762T/G1764A was most frequent and found in 75.0% of them, and combination mutation at C1653T or T1753V and A1762T/G1764A was found in 58.3% of them. To verify these mutations in genotype C1 genomes, we analysed full genomic sequences of 340 genotype C1 strains registered in GenBank and compared them with 24 Cambodian full genome sequences. We evaluated the relationship between liver disease status and these mutations in the 340 genotype C1 strains using GenBank data. The result showed that of the 340 genotype C1 strains, 47.1% had double mutation and 33.2% had combination mutation.

Analysing the mutation with liver disease status among the 340 genotype C1 strains, 16.5% of the ASC and 34.2% of CH already had combination mutation, but the combination mutation rate in LC/HCC was significantly high (95.2%). Moreover, the rate of combination mutation in 24 Cambodian C1 isolates (58.3%) was significantly higher than that in overall 340 genotype C1 strains (33.2%, p = 0.0128) and the CH alone in GenBank sequence (34.2, p = 0.241) (Table 3). Possible underlying factors causing the higher rate of mutation such as race, human genomic influence, and host immunity of the individuals exist, but the exact reasons behind them are unknown. Nevertheless, the higher rate of HBV mutation is related to the high possibility of HCC occurrence.

The double mutation at A1762T/G1764A in genotype C2 has been shown to be present at a high rate in HCC in previous reports and the combination mutation at C1653T and/or T1753V and A1762T/G1764A increases the risk of occurrence of HCC among HBV genotype C2 carriers^17,18,26–29^.

As for HBV genotype C1 genome, some reports were published; the report in the hospital-based study from South China^27^ reported a combination mutation rate at T1753V and A1762T/G1764A in CH and HCC patients of 4.8% and 14.7%, respectively. It was reported that mutations in the basal core promoter might play a synergistic role on enhancing HBV carcinogenesis. In our analysis, the rate of combination mutation of the LC/HCC patients among 340 genotype C1 strains in GenBank was also high.

It has been reported in many studies that mutations in the core promoter including Enhancer II and Basal core promoter were related to carcinogenesis. X protein is a transcriptional activator and interacts with tumor suppressive factor P53^30^. Mutation at X gene may induce uncontrolled cell proliferation^31^, but its mechanisms have not been well clarified^17^ yet. There were some cohort studies reporting that carriers with mutations developed to HCC^18,32^, but there is no report about cohorts with mutations in processes leading from ASC or CH to HCC. Further studies are needed to elucidate these mechanisms in HCC and to investigate the prognosis of HBV carriers who had combination mutation in the core promoter region by the prospective studies for follow up.

As for pre-S deletion in 340 genotype C1 strains, only one case from this study was found in 21 cases of LC/HCC (4.8%), and 21 strains were found in 340 C1 strains (3.4%) from GenBank. The rate of pre-S deletion associated with HCC was lower than that reported in genotype C2 with HCC^29,33–35^. Therefore, this deletion might not be strongly associated with HCC in genotype C1.

There were some limitations to this study. The first limitation was that we were not able to obtain the liver status of all the 340 genotype C1 strains registered in GenBank. But according to our findings in the analysis of 340 genotype C1 strains, 95% of patients with HCC had the combination mutation. These findings suggest that genotype C1 with combination mutation might be associated with a high risk of HCC.

The second limitation was that we conducted a serological pilot survey for only a small portion of residents of four regions in Siem Reap province, Cambodia. We could not reach all residents of these regions, but according to the results of this study among 24 Cambodian isolates of genotype C1, double mutation at A1762T/G1764A was observed in 78.3% and combination mutation was observed in 58.3% of the isolates. The study participants were recruited from previously conducted cross-sectional pilot studies so the liver status of the participants could not be known. Therefore, the actual correlation of mutation and liver status could not be found by this study.

Therefore, since there is some probabilities that Cambodian HBV infected residents are expected to have the risk of progression to HCC, they should be followed up with to grasp trends of their genomes over time and to take measures to prevent HCC.

## Methods

### Study design

We conducted a pilot survey among the general population in four regions in Siem Reap province, Cambodia from 2010 to 2014^21–23^. In this study, we rearranged the data for all participants (n = 626), including children, and found that the age of participants ranged from 7 to 90 years old. We then selected the participants who were positive for HBsAg and tested their serums for HBV genome sequences.

### Serological Testing of the participants

We performed the serological tests for HBV infection as follows. HBsAg was detected using reverse passive hemagglutination assays (R-PHA, Mycell II HBsAg; Institute of Immunology, Tokyo Japan) and chemiluminescent immunoassays (CLIA, Architect HBsAg QT; Abbott, Tokyo, Japan). Hepatitis B core antibody (Anti-HBc) was detected using passive hemagglutination (PHA) with Mycell anti-rHBc (Institute of Immunology, Tokyo, Japan) and CLIA with Architect HBc II (Abbott, Tokyo, Japan). Hepatitis B surface antibody (Anti- HBs) was detected using PHA with Mycell II anti-HBs (Institute of Immunology, Tokyo, Japan) and CLIA with Architect Osabu (Abbott, Tokyo, Japan).

### Subjects for HBV full-length genome sequencing

Among 35 HBsAg-positive samples, eight had insufficient amounts of virus for further analysis. Thus, 27 samples were subjected to PCR for full sequencing. Finally, we obtained 26 full genome sequences out of 27 PCR-positive samples; only one yielded a partial sequence. These 26 patients were positive for HBsAg with R-PHA method (Table 2).

### HBV full-length genome sequencing in 26 Cambodian participants

For full-length sequencing of HBV genome, we used the same primers used for PCR in the previously described method^21,36^. Briefly, we extracted 100 µL of serum for detection of HBV DNA using SMITEST EX-R&D (Genome Science Laboratories, Fukushima, Japan) and for nested PCR using Prime STAR^®^GXL polymerase (Takara Bio Inc., Shiga, Japan) with the primer set WA-L and WA-R and inner primers WA-L2 and WA-R2. For the missing portion of the circular HBV DNA, we performed nested PCR again on the extracted DNA using Prime STAR^®^GXL polymerase and the primer sets S1, S2, AS1, and AS2. Final products were sequenced using an Applied Biosystems 3730 x l DNA sequencer (Thermo Fisher Scientific K.K., Kanagawa, Japan) and a BigDye Terminator v3.1 Cycle Sequencing Kit (Applied Biosystems, Foster City, CA, USA).

### Extraction of 340 full genomes of genotype C1 registered in GenBank

To extract HBV C1 strains from GenBank, we first searched many neighbourhood genes resembling the full sequence data obtained from a Basic Local Alignment Search Tool (BLAST) screening of the National Centre for Biotechnology Information (NCBI) database. We constructed phylogenetic trees several times as shown below and determined genotype C1 strains. There were 340 registered HBV genotype C1 strains in the GenBank. We recruited all of them in this study after gathering background information and full-length genomic strains for each one.

### Genetic analysis and phylogenetic analysis

After genomic sequencing, nucleic acid analysis was performed using GENETYX-MAC version 18 software, by the UPGMA method^34^ with the 26 full genomes and HBV genotypes B1–B9 and C1–C16 strains registered in GenBank. For genotype C1, analysis was performed by the neighbour-joining method^35^ with 24 full genomes of genotype C1 and 340 HBV C1 strains selected above. Evolutionary analyses were conducted in MEGA7^37^.

### Classification of HBV genomic mutation patterns and liver statuses of participants for 340 genomes in GenBank and 24 Cambodian genomes with genotype C1

First, we classified 340 genotype C1 strains and 24 Cambodian isolates into 48 patterns by combinations of the following mutations pre-S deletion, G1613A, C1653T, T1753V, and A1762T/G1764A, Pre-C W28 stop codon, and P130 in the core region.

Second, we confirmed the backgrounds and liver statuses of the participants for the 340 genotype C1 strains registered in GenBank by investigating the original papers in which the strains were reported and the information in GenBank.

Third, we classified the isolate holders as asymptomatic carriers (ASC) in which we included blood donors (BDs), occult hepatitis B infection and general populations,  human immunodeficiency virus (HIV) and HBV co-infected patients, patients with chronic hepatitis B, patients with liver cirrhosis (LC), or patients with hepatocellular carcinoma (HCC). If there was no description of the clinical background in the GenBank registry or paper or if there was no publication with gene registration, the disease state was classified as unknown.

### Statistical analysis

Both of the proportion of double mutation and the proportion of combination mutation among ASC, CH and LC/HCC were compared using χ^2^ test for the statistical homogeneity among 3 groups and posthoc pairwise χ^2^ test with Bonferroni correction. *p* < 0.05 was considered statistically significant. All data were analysed using JMP version 11 (SAS Institute Inc., Cary, NC, USA).

### Ethical consideration

Informed consent was obtained from every participant after well explanation of the main content and purpose of the study. For participants under 18 years, informed consent was obtained from either their mother, father, or legal guardian. Informed assent was obtained from all children in the study. All specimens were de-identified, with reference only to a unique identifier. The detailed study procedure including taking informed consent for human subjects was clearly described in the study protocol. The study protocol was confirmed and approved by the Ethics Committee for Epidemiological Research of Hiroshima University, Japan (ethical no. 370-1) and the National Ethics Committee for Health Research at Ministry of Health of Cambodia (ethical no. 0085 NECHR). Additionally, we obtained permission from the National Institute of Public Health of the Ministry of Health of Cambodia (no. 1494 NIPH) for the transportation of blood samples from Cambodia to our laboratory in Hiroshima, Japan. All methods were performed in accordance with the relevant guidelines and regulation.

## Supplementary information

Mutation Patterns in 340 HBV genotype C1 strains retrieved from GenBank and 24 isolates Cambodian in the study

## Data Availability

All sequenced data of Cambodian HBV isolates are registered to GenBank via DDBJ. (Data will be open when the paper is published).
