# Supplementary material for: High possibility of hepatocarcinogenesis in HBV genotype C1 infected Cambodians is indicated by 340 HBV C1 full-genomes analysis from GenBank
Source: Sci Rep. 2019 Aug 21;9:12186. doi: 10.1038/s41598-019-48304-z (PMC6704254; doi:10.1038/s41598-019-48304-z)
Supplement: Supplementary file 1 — Mutation Patterns in 340 HBV genotype C1 strains retrieved from GenBank and 24 isolates Cambodian in the study [file 41598_2019_48304_MOESM1_ESM.pdf]

High possibility of hepatocarcinogenesis in HBV genotype C1 infected Cambodians is indicated by 340 HBV C1 full-genomes analysis from GenBank

Authors:

Channarena Chuon[1], Kazuaki Takahashi[1,2], Junko Matsuo[1], Keiko Katayama[1], Chikako Yamamoto[1], Ko Ko[1], Sirany Hok[3], Shintaro Nagashima[1], Sheikh Mohammad Fazle Akbar[2] and Junko Tanaka[1]*

Affiliations

[1]Department of Epidemiology, Infectious Diseases Control and Prevention, Graduate School of Biomedical and Health Sciences, Hiroshima University

[2]Department of Medical Sciences, Tokyo Shinagawa Hospital, Japan

[3]Ministry of Heath, Phnom Penh, Cambodia

Supplementary Table-1: Mutation Patterns in 340 HBV genotype C1 strains retrieved from GenBank and 24 isolates Cambodian in the study

	340 C1 strains in GenBank						24 Cambodian
Mutation Pattern	Sum of n (%)	ASC# n (%)	HIV+HBV n (%)	CH n (%)	LC/HCC n (%)	Unknown n (%)	Sum of n (%)
pattern 1 to 9	32 (9.41)	3 (3.1)	0	19 (12.8)	9 (42.9)	1 (1.7)	5 (20.83)
pattern 10 & 11	4 (1.18)	2 (2.1)	0	0	2 (9.5)	0	0
Pattern 12 to 23	77 (22.65)	11 (11.3)	4 (30.8)	32 (21.5)	9 (42.9)	21 (35)	9 (37.5)
Pattern 24 to 32	47 (13.82)	12 (12.4)	1 (7.7)	22 (14.8)	1 (4.8)	11 (18.3)	4 (16.67)
Pattern 33 to 48	163 (47.94)	67 (69.1)	7 (53.9)	64 (43)	0	25 (41.7)	5 (20.83)
Other	17 (5)	2 (2.1)	1 (7.7)	12 (8.1)	0	2 (3.3)	1 (4.17)
Total	340 (100)	97	13	149	21	60	24 (100)

ASC#: Asymptomatic Carrier which includes blood donors, occult hepatitis B infection and general population, HIV+HBV: Human Immunodeficiency Virus with Hepatitis B Virus, OBI: Occult Hepatitis B Infection, GP: General Population, CH: Chronic Hepatitis, LC/HCC: Liver Cirrhosis/Hepatocellular Carcinoma.
